# Combinations of Bacteriophage Are Efficacious against Multidrug-Resistant *Pseudomonas aeruginosa* and Enhance Sensitivity to Carbapenem Antibiotics

**DOI:** 10.3390/v16071000

**Published:** 2024-06-21

**Authors:** Christopher J. Kovacs, Erika M. Rapp, William R. Rankin, Sophia M. McKenzie, Brianna K. Brasko, Katherine E. Hebert, Beth A. Bachert, Andrew R. Kick, F. John Burpo, Jason C. Barnhill

**Affiliations:** 1 United States Military Academy, West Point, NY 10996, USA; christopher.kovacs@westpoint.edu (C.J.K.); rapperik@msu.edu (E.M.R.); rankin.william@mayo.edu (W.R.R.); sophia.mckenzie@usuhs.edu (S.M.M.); brianna.brasko@westpoint.edu (B.K.B.); katherine.hebert@westpoint.edu (K.E.H.); beth.bachert@westpoint.edu (B.A.B.); andrew.kick@westpoint.edu (A.R.K.); john.burpo@westpoint.edu (F.J.B.); 2 Defense Threat Reduction Agency, Fort Belvoir, VA 22060, USA

**Keywords:** bacteriophage, phage therapy, antibiotics, antibiotic resistance, *P. aeruginosa*, biofilms

## Abstract

The Gram-negative ESKAPE bacterium *Pseudomonas aeruginosa* has become a pathogen of serious concern due its extensive multi-drug resistance (MDR) profile, widespread incidences of hospital-acquired infections throughout the United States, and high occurrence in wound infections suffered by warfighters serving abroad. Bacteriophage (phage) therapy has received renewed attention as an alternative therapeutic option against recalcitrant bacterial infections, both as multi-phage cocktails and in combination with antibiotics as synergistic pairings. Environmental screening and phage enrichment has yielded three lytic viruses capable of infecting the MDR *P. aeruginosa* strain PAO1. Co-administration of each phage with the carbapenem antibiotics ertapenem, imipenem, and meropenem generated enhanced overall killing of bacteria beyond either phage or drug treatments alone. A combination cocktail of all three phages was completely inhibitory to growth, even without antibiotics. The same 3× phage cocktail also disrupted PAO1 biofilms, reducing biomass by over 75% compared to untreated biofilms. Further, the phage cocktail demonstrated broad efficacy as well, capable of infecting 33 out of 100 diverse clinical isolate strains of *P. aeruginosa*. Together, these results indicate a promising approach for designing layered medical countermeasures to potentiate antibiotic activity and possibly overcome resistance against recalcitrant, MDR bacteria such as *P. aeruginosa*. Combination therapy, either by synergistic phage-antibiotic pairings, or by phage cocktails, presents a means of controlling mutations that can allow for bacteria to gain a competitive edge.

## 1. Introduction

The continued rise of antibiotic resistance in bacteria has rendered entire classes of drugs as ineffective against certain pathogens. This is further complicated by the concurrent decline in novel antibiotic development, which has seen a vast discovery void spanning several decades [1,2,3]. Studies by both the World Health Organization and Centers for Disease Control have spotlighted this impending healthcare crisis, calling attention to pathogens of urgent concern due to their multidrug resistance (MDR) and extensive drug resistance (XDR) profiles [3,4,5]. The ESKAPE group of bacteria (*Enterococcus faecium*, *Staphylococcus aureus*, *Klebsiella pneumoniae*, *Acinetobacter baumannii*, *Pseudomonas aeruginosa*, and *Enterobacter* species) causes over 3 million infections and 60,000 deaths annually [5,6]. *P. aeruginosa* is among the most common causative agents of nosocomial infections, afflicting patients suffering from wounds, burns, and urinary tract infections, as well as lung and bronchial airway complications [7,8,9]. Chronic infections of the latter are especially debilitating for cystic fibrosis and immunocompromised patients [10,11]. Treatment options against MDR and XDR *P. aeruginosa* are becoming limited, especially as the bacterium has begun to increasingly acquire resistance to carbapenem antibiotics. Carbapenems are β-lactam drugs that inhibit bacterial cell wall synthesis and are often used as a last-line defense against Gram-negative bacteria due to their historical efficacy. Resistance to carbapenems can be traced back to the evolutionary development of carbapenemases, which hydrolyze the drug and inhibit its entrance into the bacterium [12]. The sheer magnitude of this issue is illustrated through mortality rates attributed to carbapenem-resistant *P. aeruginosa* infections—ranging from 51.2% to 95% [13]. The increasing lack of therapeutic options to treat emergent XDR bacteria necessitates the need for developing novel, effective methods to kill these debilitating pathogens.

Lytic bacteriophages (phages) are natural viral predators of bacteria that necessarily lyse and kill their host to escape. This trait has generated renewed interest in phage research towards development of new therapeutic tools to combat MDR and XDR pathogens. Various studies have suggested the effectiveness of phage therapy against MDR bacteria, including refractory *P. aeruginosa* infections of the urinary tract, prosthetic joints, and topical wounds [7,8,9]. Phages have also been shown to reinstate their host bacterium’s sensitivity to certain antibiotics, as part of the evolutionary trade-off between phage and bacterium as resistance develops. Work from the Turner Lab at Yale University demonstrated that an efflux pump of *P. aeruginosa* acts as a phage receptor. In order for the bacterium to evade the phage, mutations occurred in the OmpR component of the pump, resulting in phage resistance, yet consequentially enhancing sensitivity to multiple antibiotics [14,15]. Similar examples of this trade-off phenomenon have also been shown in MDR strains of *Escherichia coli*, *A. baumannii*, and *K. pneumoniae* [16,17,18]. Rational design of phage therapeutics may allow for cocktails of phages and antibiotics through which co-administration can result in resensitization to already available drugs.

The drug resistance profiles of *P. aeruginosa* are further compounded by the bacterium’s ability to form and persist within multispecies communities called biofilms. *P. aeruginosa* readily forms biofilms on both biotic and abiotic surfaces, allowing it to thrive on various types of medical equipment and become highly resistant to both general disinfectants and broad-spectrum antibiotics [19]. Biofilm-associated colonization of implanted medical devices is the most common reason for implant failures and often requires complicated surgical intervention to resolve [20,21,22]. Biofilms protect inhabiting bacteria from antibiotic exposure, while their proximity allows for genetic promiscuity and exchange of antibiotic resistance traits. Many of the hospital-acquired *P. aeruginosa* infections are attributed to biofilm growth, creating difficult-to-treat, recalcitrant diseases [20,22]. Phages have been shown to penetrate the extracellular polysaccharide (EPS) matrix of biofilms that antibiotics cannot and have been used to successfully disrupt biofilms of *P. aeruginosa*, *Enterococcus faecalis*, and *Proteus mirabilis* on various catheter devices [23,24,25]. In addition, phages have been used clinically to treat chronic biofilms of prosthetic joint infections by *Staphylococcus epidermidis*, methicillin-resistant *S. aureus* (MRSA), *K. pneumoniae*, and *P. aeruginosa* [8,26,27,28].

The present study describes the isolation and characterization of three phages infecting three *P. aeruginosa* strains, PAO1—PaPC1, PaWP1, and PaWP2. Phages were highly effective against *P. aeruginosa* strain PAO1 and capable of potentiating the activity of carbapenem antibiotics when administered in combination. A cocktail of all three phages was completely growth-inhibitory, while also exhibiting antibiofilm potency, providing evidence for potential effectiveness against a pathogen of urgent clinical concern.

## 2. Materials and Methods

### 2.1. Bacterial Growth

*Pseudomonas aeruginosa* strain PAO1 was cultured in tryptic soy broth (TSB; Thermo Scientific, Waltman, MA, USA) at 37 °C under constant shaking at 250 RPM. Overnight cultures (~18 h of growth) were subcultured 1:100 into fresh TSB and grown to the desired log-phase for use in experimentation. Tryptic soy agar (TSA; Thermo Scientific, Waltman, MA, USA) was used as solid medium and incubated overnight for growth at 37 °C. 

### 2.2. Bacteriophage Isolation and Propagation

**Figure 1:**
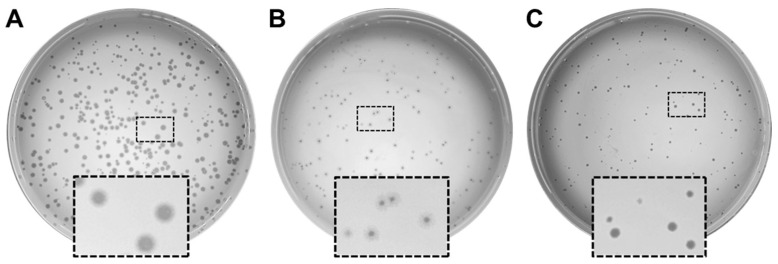
Pictographic characterization of *P. aeruginosa* PAO1 infecting phages PaPC1 (**A**), PaWP1, (**B**) and PaWP2 (**C**). Plaque morphologies for each phage are depicted.

Raw sewage samples collected from local wastewater treatment facilities were used for bacteriophage screening. Unfiltered sewage was combined with 5× TSB and log-phase *P. aeruginosa* strain PAO1 (OD600~0.4) and then incubated overnight at 37 °C under constant shaking at 250 RPM for phage enrichment. The following day, samples were centrifuged to pellet debris (10 min at 6000× *g*, 4 °C) and filter-sterilized (0.22 µm) to remove bacteria. Positive results yielded phage samples capable of lysing the target host strain, and the phages of three unique plaque morphologies were isolated (Figure 1). Phages PaPC1, PaWP1, and PaWP2 were used to prepare culture lysates by infecting growing cultures of *P. aeruginosa* strain PAO1 (at OD600 0.1) at 37 °C under constant shaking at 250 RPM until bacteria were fully lysed. Lysates were pelleted by centrifugation (10 min at 6000× *g*, 4 °C) and filter-sterilized (0.22 µm) to remove residual bacteria. Phage samples were then concentrated via centrifugation (10 min at 5000× *g*, 4 °C) using Amicon Ultra Centrifugal Filter Units (MilliporeSigma, Burlington, MA, USA) with 100K kD pore size, washed twice with SM buffer (100 mM NaCl, 8 mM MgSO_4_, 50 mM Tris-HCL, pH 7.4), and finally resuspended in SM buffer. Final phage titers were determined using the double agar overlay method for plaque enumeration (“plaque assay”) [29].

### 2.3. Whole Genome Sequencing and Annotation of Pseudomonas Phages

Genomic DNA from each phage was extracted using the Phage DNA Isolation Kit (Norgen Biotek Corp., Thorold, Ontario, Canada) and quantified by the Qubit dsDNA High Sensitivity Assay Kit (Thermo Fisher Scientific, Waltham, MA, USA). Samples were sent to CD Genomics (Shirley, NY, USA) for whole genome sequencing and annotation. Illumina sequencing libraries were prepared using the tagmentation-based and PCR-based Illumina DNA Prep kit and custom IDT 10 bp unique dual indices (UDI) with a target insert size of 320 bp. No additional DNA fragmentation or size selection steps were performed. Illumina sequencing was performed on an Illumina NovaSeq 6000 sequencer in one or more multiplexed shared-flow-cell runs, producing 2 × 151 bp paired-end reads. Demultiplexing, quality control, and adapter trimming were performed with bcl-convert1 (v4.1.5). TrimGalore software (v4.3) was utilized to remove adapters and low-quality reads from the raw Illumina data. 

De novo assembly of genomes was performed using SPAdes (v3.15.5) [30]. Quast software (v5.2.0) was applied to evaluate the statistics of contigs of the final phage assembly. Finally, Pharokka software (v1.1.0) was applied to predict the components of the phage assembly and assign putative functions to genes based on homology to known genes or protein domains [31]. The lengths and coverage for each sequence are as follows: PaPC1: 61,146 bp, 1787×; PaWP1: 106,862 bp, 169×; PaWP2: 61,933 bp, 1588×. An additional dot plot comparison between PaWP1 and its closest relative, Pseudomonas phage Zikora, was generated using the Mummer tool available through Galaxy (https://usegalaxy.org/) accessed on 24 May 2024; Galaxy Tool ID: toolshed.g2.bx.psu.edu/repos/iuc/mummer_mummer/mummer_mummer/4.0.0rc1+galaxy3.

### 2.4. Temperature and pH Stability of Phages

To determine thermostability, PaPC1, PaWP1, and PaWP2 were adjusted to ~10^6^ PFU mL^−1^ in SM buffer and then incubated at 4 °C, 25 °C, 37 °C, 45 °C, 55 °C, 65 °C and 75 °C for one hour. For pH range testing, phages were similarly prepared in SM buffer that was pH adjusted to 2.5, 4, 7, 10 and 12.5, and incubated for one hour. Phage survival was enumerated via plaque assay and is represented as mean log10 viable PFU mL^−1^ from two independent assays. No statistical differences were found when comparing control conditions (25 °C for temperature, and neutral pH) to each treatment within respective groups. 

### 2.5. Bacterial Lysis Curves 

The dose and time-kill lysis efficiency of phages was measured for PaPC1, PaWP1, and PaWP2 at various multiplicities of infections (MOIs) ranging from 10 to 0.0001 against *P. aeruginosa* strain PAO1. Early log-phase bacterial cultures (~1 × 10^7^ CFU mL^−1^) were seeded into 96-well microtiter dishes, and then mixed with phages titrated to an appropriate concentration for MOI infections (e.g., ~1 × 10^8^ PFU mL^−1^ for MOI 10, ~1 × 10^7^ PFU mL^−1^ for MOI 1, and so on). Bacterial growth was determined using a BioTek LogPhase 600 plate reader (Agilent, Santa Clara, CA, USA). Assays were performed at 37 °C under continuous orbital rotation at 250 RPM, and culture turbidity was measured at OD600, read at 20-min intervals. Lysis curves were plotted as mean ± SD OD600 values from three independent experiments, each performed with eight technical replicates. 

To determine how broadly infectious PaPC1, PaWP1, and PaWP2 were, phages were tested against a panel of 100 genetically diverse clinical isolates of *P. aeruginosa* (kindly provided by Patrick McGann from the Multidrug-Resistant Organism Repository and Surveillance Network at Walter Reed Army Institute of Research). This panel includes clinical isolates collected primarily from the US between 2003 and 2017 and was down-selected from an original collection of 3785 isolates to represent a diverse set based on multi-locus sequence typing and single nucleotide polymorphism analysis [32]. Screening involved performing bacterial growth curves (as described above) in the presence of each phage administered at MOI 1. Results were compared to growth of untreated isolates, and infectivity was determined based on appearance of lysis peaks (recorded as “+”). Changes in the outgrowth kinetics of isolates caused by phage were also noted and are indicated as “−/+” in data table. 

### 2.6. Phage–Antibiotic Combination Growth Curves

To examine phage ability to enhance sensitivity to antibiotic treatments, bacterial growth curves were created with combinations of phages and ertapenem (ERT), imipenem (IPM), and meropenem (MEM). Treatment groups consisted of 0.5× MIC of antibiotics alone (see Table S1 for antibiotic sensitivity results), PaPC1, PaWP1, and PaWP2 alone at MOI 0.001, and combinations of each phage with each antibiotic. Experiments were performed in microtiter dishes and growth of *P. aeruginosa* strain PAO1 was measured as above. Data are represented as mean ± SD OD600 values from two independent experiments, each performed with eight technical replicates. Statistical significance (*p* ≤ 0.005) was determined by pairwise comparison using Student’s *t*-test of terminal OD600 values for individual treatments versus phage plus antibiotic, within respective groups. 

### 2.7. Biofilm Disruption with PaPC1, PaWP1, and PaWP2

The ability of phages PaPC1, PaWP1, and PaWP2 to disrupt biofilms of *P. aeruginosa* strain PAO1 was assessed using established methods, with slight alterations [33]. Bacteria were grown in wells of a microtiter dish with TSB under static conditions for 24 h at 37 °C. Medium was then removed, and wells were washed gently three times with 1× Phosphate-buffered saline (PBS) to remove planktonic and loosely adhered cells. Wells were then inoculated with TSB containing either PaPC1, PaWP1, PaWP2, or a cocktail of all three phages (“3XPa”), each adjusted to ~1 × 10^9^ PFU mL^−1^, and then incubated for an additional 24 h at 37 °C. After this, medium containing phages was removed, and wells were washed gently three times with PBS. The degree of biofilm formation was measured using the crystal violet staining method [33]. Wells were filled with 200 µL of 0.1% crystal violet (*w*/*v* 30% acetic acid) and incubated at room temperature for 20 min. Stain was then removed, and wells were washed three times with dH_2_O and then allowed to air dry overnight. To quantify biofilms, 200 µL of 30% acetic acid was added to dried wells and incubated at room temperature for 20 min to solubilize stain. Wells were then read spectrophotometrically at 590 nm. Results are displayed as mean ± SD fold change compared to untreated control from three independent experiments. Statistical significance (*p* ≤ 0.005) was determined by pairwise comparison using Student’s *t*-test.

### 2.8. Data Availability

The whole genome data are available through NCBI BioProject accession number PRJNA1094968. Phage C1, WP1, and WP2 are registered as BioSamples SAMN40709553, SAMN40709608, and SAMN40709609, respectively. The annotated genome assemblies are available through NCBI GenBank under accession number PP596838 (*Pseudomonas* phage C1), PP596839 (*Pseudomonas* phage WP1), and PP596840 (*Pseudomonas* phage WP2).

## 3. Results

### 3.1. Isolation and Characterization of Phages Infecting P. aeruginosa PAO1

Bacteriophage isolation and enrichment were performed using standard methods on samples of raw sewage collected from local wastewater treatment facilities [29]. Three distinct phages were selected based on unique plaque morphologies (Figure 1). Clear, distinct plaques were observed for PaPC1 (Figure 1A), whereas PaWP1 formed a clear plaque surrounded by a hazy halo, indicative of possible depolymerase activity of phage (Figure 1B) [34]. Plaques formed by PaWP2 were clear, yet smaller than those formed by PaPC1 (Figure 1C). 

### 3.2. Genome Characterization and Genome Similarity of PaPC1, PaWP1, and PaWP2 to Existing Phages

The genomes of PaPC1 and PaWP2 exhibited similar lengths and GC content: 61,146 bp and 63.35% for PaPC1, and 61,933 bp and 63.41% for PaWP2. The genome of PaWP1 was much larger with a lower GC content, 106,862 bp and 56.58%. The number of coding sequences (CDS) for PaPC1, PaWP1, and PaWP2, as determined by SPAdes prediction, were 90, 320, and 97, respectively. Table 1 shows the number and types of functional annotations assigned to CDSs from each genome corresponding to the PHROG (prokaryotic virus remote homologous groups) library [34]. Functional categories identified in all three phages include connector, DNA, RNA and nucleotide metabolism, head and packaging, lysis, tail, and unknown function. PaWP2 harbored a unique CDS corresponding to moron, auxiliary metabolic gene, and host takeover. More than half of the CDSs predicted in all three phages harbor unknown functions. Notably, no hits were identified in the Virulence Factor Database (VFDB) or the Comprehensive Antibiotic Resistance Database (CARD), indicating the absence of virulence factors or antibiotic resistance elements that might disqualify these phages from therapeutic consideration. To predict each phage type, we utilized phageAI, a machine learning model developed to assist in classification of phages [35]. Both PaPC1 and PaWP2 were identified as virulent (lytic) phages with 95.7% and 92.01% accuracy, respectively. PaWP1 was predicted as a temperate phage, albeit with very low accuracy, 64.17%, with the caveat that the prediction was not reliable due to the lack of similar phages available. However, phage Zikora, the closest relative to PaWP1, was predicted to be a virulent phage with 96.15% accuracy. In agreement with these predictions, no integrase or excision genes were identified among all three phages, while lysis genes were present in all three phages (three lysis genes in PaPC1 and PaWP2, one lysis gene in PaWP1; Table 1). Based on these predictive attributes, we conclude that all three of our newly isolated phages are purely lytic.

Nucleotide BLAST searches of completed genomes for PaPC1, PaWP1, and PaWP2 were performed to determine similarity to existing genomes. The closest hit for PaPC1 was Pseudomonas phage Aergia, with a query cover of 91% and 97.02% identity (GenBank accession: OR805291.1). The closest hit for PaWP1 was Pseudomonas phage Zikora (93.09% identity, 98% query cover; GenBank accession: MW557846.1). Notably, a large section of the phage Zikora genome was found to be duplicated in PaWP1. Positions 5073–57,884 and 5073–43,437 of Zikora align to positions 51,520–104,452 and 1–38,392 of PaWP1, spanning ~53 kb and ~38 kb, respectively. To confirm the duplication, a whole genome dot plot was generated from the FASTA sequences using Mummer v4.0 [36]. A nearly continuous diagonal line was observed, indicating overall similarity across the genome, with an additional diagonal running parallel to the main diagonal, indicating a repeated region on a different part of the sequence, corresponding to the aforementioned locations (Figure S2). The closest hit for PaWP2 was Pseudomonas phage Chuck (98.19% identity, 97% query cover; GenBank accession: OQ992557.1). PaWP1 likely represents a true novel phage, since its percent similarity to existing phage is less than 95%, following the criterion established by the International Committee on Taxonomy of Viruses (ICTV) [37]. Based on BLASTn analysis, both PaPC1 and PaWP2 phages belong to the class Caudoviricetes, subfamily Rabinowitzvirinae, genus Yuavirus, while PaWP1 belongs to the class Caudoviricetes, genus Pbunavirus. All three phages are expected to be linear double-stranded DNA (dsDNA) viruses with head–tail morphology, as evidenced by BLASTn taxonomy and Pharokka annotation.

**Table 1:** Genome features of PAO1 phages.

Features	PaC1Φ	PaWP1Φ	PaWP2Φ
CDS	90	320	97
connector	4	1	4
DNA, RNA, and nucleotide metabolism	12	11	10
head and packaging	10	26	10
integration and excision	0	0	0
lysis	3	1	3
moron, auxiliary metabolic gene and host takeover	0	0	1
other	1	5	1
Tail	8	27	9
transcription regulation	0	0	0
unknown function	52	249	59
tRNAs	0	0	0
CRISPRs	0	0	0
tmRNAs	0	0	0
† VFDB_Virulence_Factors	0	0	0
‡ CARD_AMR_Genes	0	0	0

† VFDB: Virulence Factor Database [38]; ‡ CARD: Comprehensive Antibiotic Resistance Database [39].

### 3.3. PaPC1, PaWP1, and PaWP2 Demonstrate Thermostability and pH Tolerance

**Figure 2:**
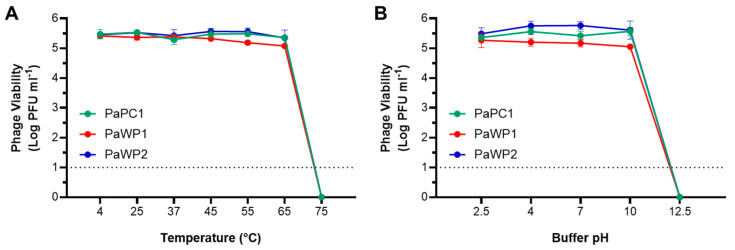
Bacteriophage thermostability (**A**) and pH tolerance (**B**) was assessed for PaPC1 (**•**), PaWP1 (**•**) and PaWP2 (**•**). Phages were incubated for 1 h at indicated conditions and then enumerated via plaque assay using the double agar overlay method. Data are representative of two independent experiments and displayed as mean ± SD log PFU mL^−1^ of viable phages. Hashed line indicates limit of detection (no phages were recovered for either 75 °C or pH 12.5).

Phage stability was assessed across a range of temperatures and pH values. Testing at 4 °C, 25 °C, 37 °C, 45 °C, 55 °C, and 65 °C had no inhibitory effect on any of the three phages, with consistent titers enumerated following 1 h incubation for each (Figure 2A). No viable phages were recovered after incubation at 75 °C. All three phages tolerated acidic conditions, with phage titers from pH 2.5 and 4 comparable to those from neutral conditions. Mild alkaline exposure at pH 10 was also inconsequential; however, none of the three phages survived exposure to pH 12.5 for an hour (Figure 2B). It is worth noting that the limit of detection was 10 or fewer phages due to experimental design, so it is not clear if either 75 °C or pH 12.5 were capable of complete kill. Nonetheless, these results demonstrate that PaPC1, PaWP1, and PaWP2 have wide thermostability and pH tolerance.

### 3.4. Lytic Infection Profile of Phages PaPC1, PaWP1, and PaWP2

**Figure 3:**
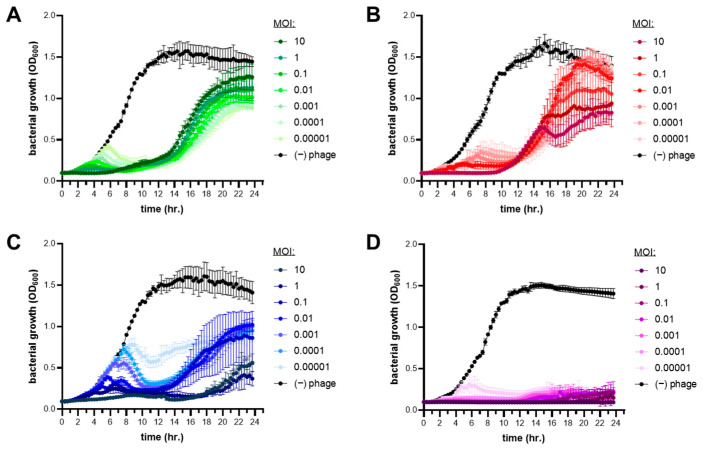
Bacterial lysis of *P. aeruginosa* PAO1 by phages PaPC1 (**A**), PaWP1 (**B**), PaWP2 (**C**), and in combination as a cocktail (**D**). Bacteria were grown in the presence of varying multiplicities of infection (MOIs) of phages for 24 h and measured spectrophotometrically at optical density (OD) 600 nm. Data are representative of three independent experiments and displayed as mean ± SD.

To determine the lytic profiles for PaPC1, PaWP1, and PaWP2, lysis curves were created by infecting *P. aeruginosa* PAO1 cultures with various multiplicities of infections (MOI) of phages and grown for 24 h. The MOI ranged from 10–0.0001 and in all cases were growth-inhibitory in a dose-dependent manner (Figure 3). Despite the lytic efficacy of single phage treatments, bacteria were capable of developing phage resistance under all MOIs for each of the three phages as evidenced by the delayed increase of OD600 values at ~12 h of incubation (Figure 3). While the acquired resistance allowed *P. aeruginosa* PAO1 to escape phage infection, the mutants that arose from PaPC1, PaWP1, and PaWP2 treatment were incapable of reaching the same maximal cell densities as the untreated wild type. Interestingly, multiple lysis peaks were observed in growth curves of bacteria exposed to both PaWP1 and PaWP2, suggesting successive mutations in the culture to evade phage infection. A phage cocktail consisting of equal parts PaPC1, PaWP1, and PaWP2 in mixture was inhibitory to growth against *P. aeruginosa* PAO1 and was able to prevent the development of phage resistance for all tested MOIs out to 24 h (Figure 3D).

### 3.5. Combinations of Phages with Antibiotics Enhances Carbapenem Sensitivity of P. aeruginosa PAO1

**Figure 4:**
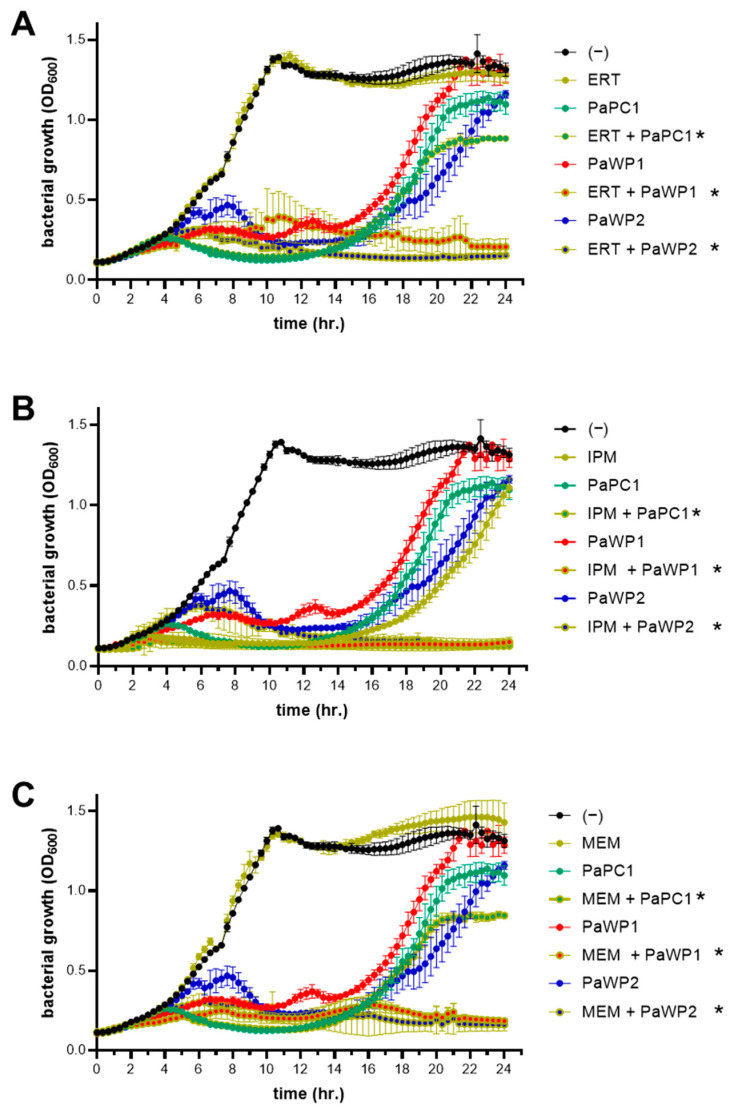
The drug potentiating effects of phages were determined in combination with antibiotics. *P. aeruginosa* PAO1 bacteria was grown in the presence of ertapenem (ERT; (**A**)), imipenem (IPM; (**B**)), meropenem (MEM; (**C**)), PaPC1, PaWP1, and PaWP2 either alone (closed circles) or in combinations (encircled in gold) for 24 h and measured spectrophotometrically at optical density (OD) 600 nm. Phages were all applied at an MOI of 0.001, while antibiotics were used at 0.5× MIC. Data are representative of two independent experiments and displayed as mean ± SD. * *p* ≤ 0.005, combinations versus comparable single treatments at terminal data points.

The antibiotic resistance profile of *P. aeruginosa* PAO1 was confirmed by minimum inhibitory concentration (MIC) testing in broth microdilutions and summarized in Table S1. Experiments confirmed multidrug resistance across each class of antibiotics, notably as well among the antipseudomonal carbapenems tested—ertapenem (ERT), imipenem (IPM), and meropenem (MEM). To assess the ability of phages to complement these drugs and restore sensitivity, subinhibitory concentrations of PaPC1, PaWP1, and PaWP2 (MOI 0.001) were each combined with 0.5× MIC of ERT, IPM, and MEM and administered to cultures of *P. aeruginosa* PAO1 while growth was measured over 24 h (Figure 4). Bacterial growth in the presence of both ERT and MEM at 0.5× MIC was nearly identical to the untreated control, whereas 0.5× MIC IPM inhibited bacterial growth for approximately 14 h, after which the culture was able to grow exponentially. When the same antibiotic concentrations were combined with phages, bacterial outgrowth was substantially reduced. Combinations of ERT (Figure 4A), IPM (Figure 4B) and MEM (Figure 4C) with both PaWP1 and PaWP2 were most efficient, resulting in complete inhibition of outgrowth after 24 h. PaPC1 when combined with IPM (Figure 4B) had similar effects, while together with ERT (Figure 4A) and MEM (Figure 4C) reduced bacterial outgrowth greater than either phage or respective antibiotic alone. Comparing the OD600 measurement at the terminal timepoint (e.g., the final culture density) all combination treatments of PaPC1, PaWP1, and PaWP2 with ERT, IPM, and MEM were significantly reduced in comparison to single agent treatments within respective groups (*p* ≤ 0.005).

### 3.6. PaPC1, PaWP1, and PaWP2 Phages Disrupt Biofilms of P. aeruginosa PAO1

**Figure 5:**
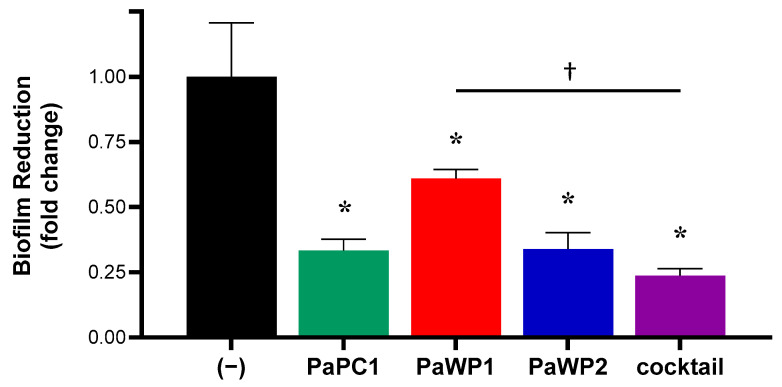
Disruption of *P. aeruginosa* PAO1 biofilms by isolated phages. Biofilms were grown in a microtiter dish for 24 h and then treated with ~1 × 10^8^ PFU mL^−1^ for an additional 24 h and quantified by crystal violet staining of adhered cells. Data are representative of three independent experiments and displayed as mean ± SD fold change of spectrophotometric readings at 590 nm. * *p* ≤ 0.005, compared to untreated control; † *p* ≤ 0.005.

*P. aeruginosa* can form difficult-to-treat biofilms on both biotic and abiotic surfaces, causing recalcitrant infections in wounds and the lungs of cystic fibrosis patients, as well as on implanted medical devices [20,21,22]. To assess the antibiofilm activities of PaPC1, PaWP1, and PaWP2, *P. aeruginosa* PAO1 bacteria were grown in polystyrene microtiter plates for 24 h, followed by phage treatments for an additional 24 h. Adherent cells were stained with crystal violet and biomass was quantified spectrophotometrically at optical density 590 nm. Measured biofilms were significantly reduced by 66.7%, 39.1% and 62.9% following incubation with phages PaPC1, PaWP1, and PaWP2, respectively (*p* ≤ 0.005; Figure 5). The cocktail of all three phages produced even greater antibiofilm effects, significantly reducing the biomass by more than 75% (*p* ≤ 0.005).

## 4. Discussion

As bacteria continue to develop and acquire means to resist antibiotic treatment, phages that can effectively kill MDR bacteria have become sought after for the development of alternative therapeutic options. We aimed to isolate novel phages against *P. aeruginosa*, a member of the ESKAPE group of bacteria, and successfully identified three unique viruses with lytic activity against strain PAO1. Phages PaPC1, PaWP1, and PaWP2 displayed clear, distinct plaque formation, suggestive of lytic phage activity. Plaques formed by PaWP1 were surrounded by a hazy halo, a morphology that has been attributed to depolymerase activity of the phage baseplate [40]. Phage depolymerases have been suggested to steer host strain recognition and, by degradation of capsular polysaccharides, can expose secondary receptors to phage binding [40,41]. In addition, isolated depolymerase enzymes from phages have been shown to synergize with antibiotics and degrade the extracellular matrix of biofilms [42,43]. It remains to be seen to what extent PaWP1 can specifically degrade *P. aeruginosa* polysaccharides, and efforts are underway to identify and isolate the depolymerase enzyme. 

Whole genome sequencing of PaPC1, PaWP1, and PaWP2 revealed several interesting observations. PaWP1 represents a novel phage, with a percent similarity of 93.09% to the top BLAST hit, phage Zikora, lower than the 95% cutoff for a novel phage [37]. This genome was also much larger than PaPC1 and PaWP2. The genome sequence of Pseudomonas phage Zikora was reported as a new member of the *Pbunavirus* genus, with 92 ORFs identified [44]. While Zikora was reported as a temperate phage, our data show that PaWP1 is a purely lytic phage, with 320 CDSs identified, many of which have unknown functions. Future studies will investigate the functions of these putative proteins. 

All three phages returned no hits for virulence factors or antibiotic resistance genes as assessed by Pharokka annotation and comparison against the VFDB and CARD databases. Moreover, the distinct plaque formations of each phage, along with the lack of integrase or excision genes and presence of lytic genes, verifies these phages as purely lytic. The lack of problematic virulence and resistance genes, along with the lytic phenotype are features desirable for a therapeutic phage, indicating PaPC1, PaWP1, and PaWP2 would be suitable for therapeutic use.

Over half of the CDSs predicted in all three phages have unknown functions, demonstrating the lack of information available about these and other sequenced phage genomes. Significant work is required to determine the functions of these proteins, which may answer important questions about the proteins involved in recognizing host cells and facilitating viral entry, as well as the viral life cycle. 

The favorable lysis curves for each phage represent strong lytic activity, although resistance to infection did develop within the 24-h exposure period. The resulting mutant populations appeared to have altered fitness, however, as their ability to reach maximal culture density was severely reduced, as compared to untreated controls. While the development of phage resistance is not ideal, if it also causes deficient bacterial fitness, then those surviving cells may be more susceptible to other means of killing, such as by immune clearance, or antibiotic treatment [1,14,15,16,17]. When PaPC1, PaWP1, and PaWP2 were combined as a three-phage cocktail, bacterial outgrowth was completely inhibited, suggesting that the selective pressure from all three phages could sufficiently suppress resistance from developing. 

Phage–antibiotic synergistic pairings have been well documented, and combining the two antimicrobials is the favored approach in clinical phage therapy applications. Carbapenem antibiotics have strong potency against Gram-negative bacteria, although their effectiveness is waning as bacteria, including *P. aeruginosa*, acquire carbapenemase enzymes to degrade the drug [12,13]. To assess the drug-potentiating capacity of PaPC1, PaWP1, and PaWP2, phages were combined with subinhibitory concentrations of ertapenem, imipenem, and meropenem. In all cases, the combinations were more effective than singular treatments, substantially reducing outgrowth kinetics, as well as maximal culture density. Moreover, combinations that included PaWP1 and PaWP2 were completely growth-inhibitory by 24 h, as well as for PaPC1 combined with imipenem. If bacterial resistance to phages did indeed result in a fitness trade-off, it is interesting to speculate if this is tied directly to tolerance of carbapenems. *P. aeruginosa* strain PAO1 does not produce carbapenemases, so this likely occurs through an alternative mechanism besides canonical resistance. As aforementioned, phage depolymerases can contribute to antibiotic sensitization [42]. The degradation of cell wall polysaccharides can dramatically impact bacterial fitness, leading to impairments in peptidoglycan turnover and cellular division [45,46]. If PaWP1 exhibits depolymerase activities, one possible explanation for the enhanced sensitivities may be that the cell wall degradation caused by the phage allows for better access of the β-lactam carbapenems to their peptidoglycan-inhibiting target. Additional studies need to be performed to test this hypothesis, and the degree to which phages PaPC1 and PaWP2 can perform the same activity (if at all) still needs to be investigated. 

Chronic infections caused by *P. aeruginosa* are often associated with the formation of biofilms, which the bacterium can readily produce on both biotic and abiotic surfaces. Phages have been effective at removal of *P. aeruginosa* biofilms on catheters, endotracheal tubes, topical wounds, as well as in ex vivo studies on fibroblasts using clinical isolates from cystic fibrosis patients [9,11,23,47]. In addition, bacteriophage therapy has had documented clinical success against *P. aeruginosa* biofilm infections of prosthetic joints, an aortic graft, and in treatment of a cystic fibrosis patient with chronic lung infection [8,48,49]. Treatment of *P. aeruginosa* strain PAO1 biofilms with PaPC1, PaWP1, and PaWP2 resulted in significantly reduced biomass, with even greater reduction observed for a cocktail of all three phages. The combinatorial effects of the phage cocktail on biofilms were consistent with results from exposure to planktonic culture. While plaque morphology for PaWP1 was suggestive of possible depolymerase activity, it is worth noting that this phage displayed the least amount of antibiofilm activity in single treatment. Besides polysaccharides, the biofilm EPS is also largely composed of extracellular proteins and eDNA [50]. A single exonuclease gene was found in both PaPC1 (locus tag PC1_019) and PaWP2 (locus tag WP2_057), while PaWP1 appeared to lack exonuclease genes. It is possible that PaWP1 produced plaques with halos that were remnants of the biofilm the phage was unable to clear, while PaPC1 and PaWP2 produced clear plaques as they were able to clear both bacterial cells and biofilm via exonuclease activity. This is also consistent with the decreased antibiofilm activity observed for PaWP1 in Figure 5, while PaPC1 and PaWP2 both demonstrated superior antibiofilm activities in isolation. The additive effect of matrix degradation coupled with lytic phage activity may provide better diffusion of phages with expanded efficacy. Similar results have been reported for phage cocktails targeting biofilms of clinical isolate *P. aeruginosa* strains [51].

## 5. Conclusions

Phages PaPC1, PaWP1, and PaWP2 have definitive lytic activity and can improve the efficacy of other antimicrobials when used in combination. Cocktails of these three phages completely prevent growth and have potent antibiofilm properties against *P. aeruginosa* strain PAO1, while also being broadly efficacious against numerous clinical isolate MDR *P. aeruginosa* stains. Taken together, these phages provide a platform for development of new and effective layered medical countermeasures to combat against life-threatening infections.

## Data Availability

Bacteriophage details available at https://www.ncbi.nlm.nih.gov/genbank/ (data released on 14 May 2024) under accession numbers: BankIt2812963 Pseudomonas_phage_PC1: PP596838; BankIt2812963 Pseudomonas_phage_WP1: PP596839; BankIt2812963 Pseudomonas_phage_WP2: PP596840.

## Supplementary Materials

The following supporting information can be downloaded at: https://www.mdpi.com/article/10.3390/v16071000/s1, Table S1. Minimum inhibitory concentration testing; Figure S1. Phage screening for broad infectivity across 100 clinical isolate strains of *P. aeruginosa*; Figure S2. Whole genome dot plot comparison of *Pseudomonas* phage Zikora and PaWP1 [52].
