# Supplementary material for: Combinations of Bacteriophage Are Efficacious against Multidrug-Resistant Pseudomonas aeruginosa and Enhance Sensitivity to Carbapenem Antibiotics
Source: Viruses. 2024 Jun 21;16(7):1000. doi: 10.3390/v16071000 (PMC11281517; doi:10.3390/v16071000)
Supplement: Supplementary file 1 [file viruses-16-01000-s001.zip › viruses-3004776-supplementary.pdf]

SUPPLEMENTAL INFORMATION

Supplemental Methods

S2.1. Minimum inhibitory concentration testing

MIC testing was performed by broth microdilution method as per Clinical and Laboratory Standards Institute guidelines to assess antimicrobial efficacy of avibactam, aztreonam, cefepime, ceftazidime, ceftriaxome, D-cycloserine, oxacillin, tazobactam/piperacillin, sulbactam/ampicillin, ertapenem, imipenem, meropenem, ciprofloxacin, levofloxacin, novobiocin, rifampicin, sulfadiazine, sulfamethoxazole/trimethoprim, amikacin, spectinomycin, tobromycin, streptomycin, kanamycin, linezolid, chloramphenicol, erythromycin, azithromycin, clindamycin, tetracycline, and tigecycline against *P. aeruginosa* strain PAO1.[1] Briefly, two-fold serial dilutions of test article were made across wells of a 96-well microtiter plate in 100 µl of TSB medium, to which 100 µl of TSB containing ~1.0 x 10^5 cells were added. Plates were incubated overnight at 37°C in static conditions. MIC values were determined as the lowest concentration within a dilution scheme that lacked evident growth.

S2.2. Phage screening for broad infectivity across 100 clinical isolate strains of P. aeruginosa

To determine how broadly infectious PaPC1, PaWP1, and PaWP2 were, phages were tested against a panel of 100 clinical isolates of *P. aeruginosa* (kindly provided by Patrick McGann from the Multidrug-Resistant Organism Repository and Surveillance Network at Walter Reed Army Institute of Research). Screening involved performing bacterial growth curves (as described in Materials and Methods section) in presence of each phage administered at MOI 1. Results were compared to growth of untreated isolates, and infectivity was determined based on appearance of lysis peaks (recorded as "+").

S2.3. Whole genome dot plot comparison of Pseudomonas phage zikora and PaWP1.

The closest hit for PaWP1 was *Pseudomonas* phage Zikora (93.09% identity, 98% query cover; GenBank accession: MW557846.1). Notably, a large section of the phage Zikora genome was found to be duplicated in PaWP1; positions 5,073- 57,884 and 5073- 43,437 of Zikora align to positions 51,520- 104,452 and 1- 38,392 of PaWP1, respectively. To confirm the duplication, a whole-genome dot plot was generated from the FASTA sequences. A nearly continuous diagonal line was observed, indicating overall similarity across the genome, with an additional diagonal running parallel to the main diagonal, indicating a repeated region on a different part of the sequence, corresponding to the aforementioned locations.

Supplemental Data

Table S1. MIC values (μg ml^{-1}) for *P. aeruginosa* strain PAO1

Cell wall inhibitors

AVB	AZT	FEP	CAZ	CRO	CYC	OXA	TZP	SAM	ERT	IPM	MEM
>256	2	0.5	2	8	>256	>256	2	256	32	4	8

Nucleic acid synthesis inhibitors

CIP	LVF	NOV	RIF	SFZ	SXT
1	4	>256	16	>256	256

Protein synthesis inhibitors

AMK	SPT	TOB	STR	KAN	LNZ	CHL	ERY	AZM	CDC	TET	TGC
2	256	0.5	16	64	>256	>256	128	32	>256	256	16

AVB, avibactam; AZT aztreonam; FEP, cefepime; CAZ, ceftazidime; CRO, ceftriaxome; CYC, D-cycloserine; OXA, oxacillin; TZP, tazobactam/piperacillin; SAM, sulbactam/ampicillin; ERT, ertepenem; IPM, imipenem; MEM, meropenem; CIP, ciprofloxacin; LVF, levofloxacin; NOV, novobiocin; RIF, rifampicin; SFX, sulfadiazine; SXT, sulfamethoxazole/ trimethoprim; AMK, amikacin; SPT, spectinomycin; TOB, tobromycin; STR, streptomycin; KAN, kanamycin; LNZ, linezolid; CHL, chloramphenicol; ERY, erythromycin; AZM, azithromycin; CDC, clindamycin; TET, tetracycline; TGC, tigecycline

	PaPC1	PaWP1	PaWP2
Pa Div-1	-	-	-
Pa Div-2	-	+	-
Pa Div-3	-	+	-
Pa Div-4	+	+	+
Pa Div-5	-	+	-
Pa Div-6	-	-	-
Pa Div-7	-	+	+
Pa Div-8	-	+	-
Pa Div-9	-	-	-
Pa Div-10	-	-	-
Pa Div-11	-	-	+
Pa Div-12	-	+	-
Pa Div-13	-	-	-
Pa Div-14	+	-	+
Pa Div-15	-	+	-
Pa Div-16	+	+	-
Pa Div-17	-	+	-
Pa Div-18	-	-	-
Pa Div-19	-	+	-
Pa Div-20	-	-	-
Pa Div-21	-	-	-
Pa Div-22	-	-	-
Pa Div-23	-	-	+
Pa Div-24	-	+	-
Pa Div-25	-	+	+

	PaPC1	PaWP1	PaWP2
Pa Div-26	-	-	-
Pa Div-27	-	-	-
Pa Div-28	-	+	+
Pa Div-29	-	-	-
Pa Div-30	-	-	-
Pa Div-31	-	-	-
Pa Div-32	-	-	-
Pa Div-33	-	-	-
Pa Div-34	-	-	-
Pa Div-35	-	-	-
Pa Div-36	-	-	-
Pa Div-37	-	-	-
Pa Div-38	-	+	-
Pa Div-39	-	+	+
Pa Div-40	-	-	-
Pa Div-41	-	-	-
Pa Div-42	-	-	-
Pa Div-43	-	+	-
Pa Div-44	-	-	-
Pa Div-45	-	-	-
Pa Div-46	-	-	-
Pa Div-47	-	-	-
Pa Div-48	-	-	-
Pa Div-49	-	-	-
Pa Div-50	-	-	-

	PaPC1	PaWP1	PaWP2
Pa Div-51	-	+	+
Pa Div-52	-	-	-
Pa Div-53	-	-	-
Pa Div-54	-	-	-
Pa Div-55	-	+	-
Pa Div-56	-	-	-
Pa Div-57	-	-	-
Pa Div-58	-	+	+
Pa Div-59	-	-	-
Pa Div-60	-	-	-
Pa Div-61	-	-	-
Pa Div-62	-	-	-
Pa Div-63	-	-	-
Pa Div-64	-	-	-
Pa Div-65	-	-	-
Pa Div-66	-	+	-
Pa Div-67	-	-	-
Pa Div-68	-	+	+
Pa Div-69	-	-	-
Pa Div-70	-	-	-
Pa Div-71	-	+	-
Pa Div-72	-	-	-
Pa Div-73	-	-	-
Pa Div-74	-	+	+
Pa Div-75	-	-	-

	PaPC1	PaWP1	PaWP2
Pa Div-76	-	+	+
Pa Div-77	-	-	+
Pa Div-78	-	-	-
Pa Div-79	-	-	-
Pa Div-80	-	-	-
Pa Div-81	-	-	-
Pa Div-82	-	-	-
Pa Div-83	-	+	-
Pa Div-84	-	+	-
Pa Div-85	-	-	-
Pa Div-86	-	-	-
Pa Div-87	-	-	-
Pa Div-88	-	+	-
Pa Div-89	-	-	-
Pa Div-90	-	+	-
Pa Div-91	+	+	+
Pa Div-92	-	+	-
Pa Div-93	-	-	-
Pa Div-94	-	-	-
Pa Div-95	-	-	-
Pa Div-96	+	-	-
Pa Div-97	-	+	-
Pa Div-98	-	-	-
Pa Div-99	-	-	-
Pa Div-100	-	-	-

Figure S1. Visual representation of phage infectivity across MRSN diversity panel of clinical isolate *P. aeruginosa* strains for PaPC1, PaWP1, and PaWP2. Positive infection is indicated by green "+" while red "-" represents no infection.

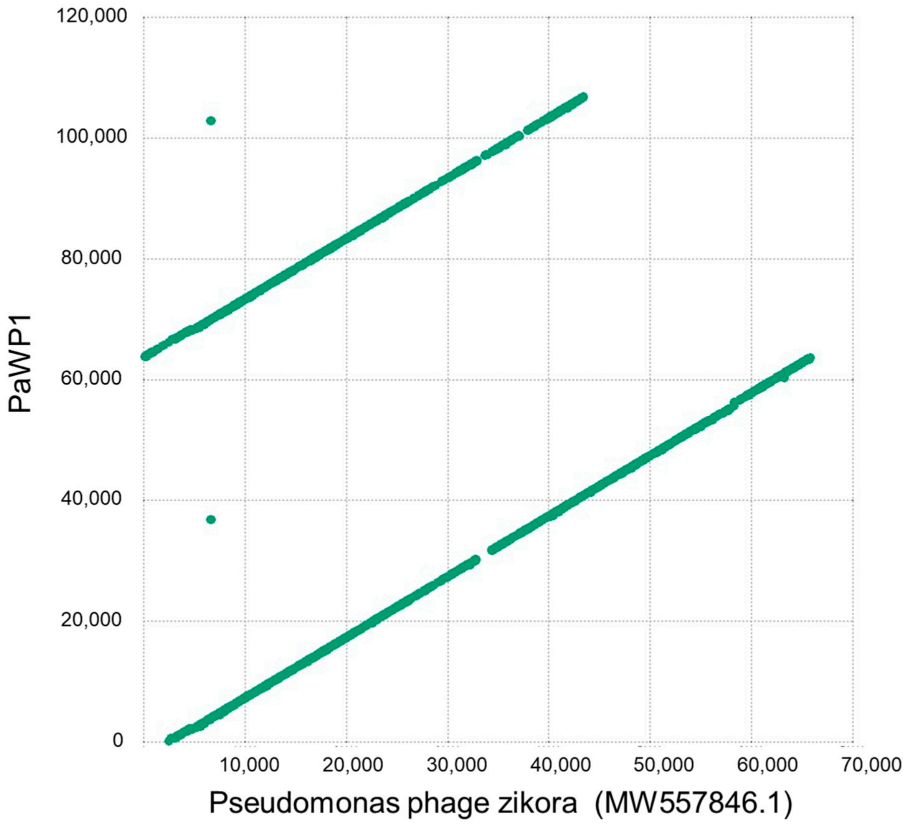

Figure S2. Whole genome dot plot comparison of *Pseudomonas* phage zikora and PaWP1. Fasta sequences for *Pseudomonas* phage Zikora (Genbank accession MW557846.1), genome length 65,837 bp, and PaWP1, genome length 106,862 bp, were uploaded to Galaxy (Galaxy (usegalaxy.org)). Mummer was utilized to generate a dot plot of the aligned genomes to assess their synteny (toolshed.g2.bx.psu.edu/repos/iuc/mummer_mummer/mummer_mummer/4.0.0rc1+galaxy3). Phage Zikora is represented on the x-axis and PaWP1 is represented on the y-axis; axis values denote nucleotide positions along each genome.

SUPPLEMENTAL REFERENCES

1. Clinical Laboratory Standards Institute. 2018. Methods for dilution antimicrobial susceptibility tests for bacteria that grow aerobically; approved standard—10th ed. M07-A11. Clinical and Laboratory Standards Institute, Wayne, PA.
